# Effects of Different Inhalation Therapy on Ventilator-Associated Pneumonia in Ventilated COVID-19 Patients: A Randomized Controlled Trial

**DOI:** 10.3390/microorganisms10061118

**Published:** 2022-05-28

**Authors:** Nikola Delić, Andrija Matetic, Josipa Domjanović, Toni Kljaković-Gašpić, Lenko Šarić, Darko Ilić, Svjetlana Došenović, Josipa Domazet, Ruben Kovač, Frane Runjić, Sanda Stojanović Stipić, Božidar Duplančić

**Affiliations:** 1 Department of Anesthesiology, University Hospital of Split, Spinčićeva 1, 21000 Split, Croatia; tkljakgas@kbsplit.hr (T.K.-G.); lsaric@kbsplit.hr (L.Š.); dilic@kbsplit.hr (D.I.); sdosenovic@kbsplit.hr (S.D.); jdomazet@kbsplit.hr (J.D.); rkovac@kbsplit.hr (R.K.); sastojanovic@kbsplit.hr (S.S.S.); bduplanc@kbsplit.hr (B.D.); 2 Department of Cardiology, University Hospital of Split, 21000 Split, Croatia; andrija.matetic@gmail.com (A.M.); frane.runjic@gmail.com (F.R.); 3 Department of Nephrology, University Hospital of Split, 21000 Split, Croatia; josipa.domjanovic@gmail.com

**Keywords:** inhalation therapy, mechanical ventilation, COVID-19

## Abstract

The effect of routine inhalation therapy on ventilator-associated pneumonia (VAP) in mechanically ventilated patients with the coronavirus disease (COVID-19) has not been well-defined. This randomized controlled trial included 175 eligible adult patients with COVID-19 who were treated with mechanical ventilation at the University Hospital of Split between October 2020 and June 2021. Patients were randomized and allocated to a control group (no routine inhalation) or one of the treatment arms (inhalation of N-acetylcysteine; 5% saline solution; or 8.4% sodium bicarbonate). The primary outcome was the incidence of VAP, while secondary outcomes included all-cause mortality. Routine inhalation therapy had no effect on the incidence of bacterial or fungal VAP nor on all-cause mortality (*p* > 0.05). Secondary analyses revealed a significant reduction of Gram-positive and methicillin-resistant *Staphylococcus aureus* (MRSA) VAP in the treatment groups. Specifically, the bicarbonate group had a statistically significantly lower incidence of Gram-positive bacterial VAP (4.8%), followed by the N-acetylcysteine group (10.3%), 5% saline group (19.0%), and control group (34.6%; *p* = 0.001). This difference was driven by a lower incidence of MRSA VAP in the bicarbonate group (2.4%), followed by the N-acetylcysteine group (7.7%), 5% saline group (14.3%), and control group (34.6%; *p* < 0.001). Longer duration of ventilator therapy was the only significant, independent predictor of any bacterial or fungal VAP in the multivariate analysis (aOR 1.14, 95% CI 1.01–1.29, *p* = 0.038 and aOR 1.05, 95% CI 1.01–1.10, *p* = 0.028, respectively). In conclusion, inhalation therapy had no effect on the overall VAP incidence or all-cause mortality. Further studies should explore the secondary findings of this study such as the reduction of Gram-positive or MRSA-caused VAP in treated patients.

## 1. Introduction

Novel coronavirus disease (COVID-19) emerged as a global health burden with numerous health consequences. The severity of symptomatic COVID-19 ranges from mild to critical, while critical patients develop respiratory failure, acute respiratory distress syndrome (ARDS), septic shock, and/or multiple organ dysfunction in approximately 5% of cases [1]. Critically ill patients with COVID-19 require particular care and additional health resources, leading to a substantial load on the overall health system.

Basic principles of critical care management include hemodynamic and respiratory stabilization, along with principal disease assessment, treatment of comorbidities, and prevention of nosocomial complications [2]. These principles also apply to the management of COVID-19 patients, but the high burden of resistant co-infections in critically ill COVID-19 patients represents a substantial health problem. Ventilator-associated pneumonia (VAP) is an important cause of morbidity and mortality in patients with ARDS of different causes and has a strong association with COVID-19 [3,4]. Various preventive measures have been suggested and implemented, including weaning protocols, minimized sedation, reverse transfusion bed position, control of the endotracheal cuff pressure, oral care, and ventilator circuit maintenance [5].

Different pathophysiological factors can mediate the development of VAP. First, airway irritation and microbiological interference induce protective mucus secretion. However, impaired host mechanisms could potentiate the inflammatory cascade, impair the feedback loop, and increase pathologic hypersecretion with airway obstruction [6]. The use of local mucolytics in mechanically ventilated patients could, theoretically, interfere with this pathophysiological process, but there are no contemporary guidelines on their routine utilization [6]. In line with that, the lack of literature discourages any recommendations on their routine use in critically ill patients with COVID-19. Nevertheless, different centers have reported the use of various mucolytic agents, such as N-acetylcysteine, hypertonic 5% saline, and 8.4% sodium bicarbonate, without significant, associated adverse events [7,8,9].

Therefore, this randomized controlled trial aimed to determine the effects of inhalation therapy (N-acetylcysteine, hypertonic 5% saline, and 8.4% sodium bicarbonate) on the incidence of bacterial or fungal VAP in mechanically ventilated COVID-19 patients. In addition, it aimed to determine any differences in all-cause mortality between trial groups. Finally, it aimed to explore any microorganism-based differences for hypothesis-generating purposes.

## 2. Materials and Methods

### 2.1. The Rationale for the Research

Mechanical ventilation is a life-saving treatment in respiratory insufficient patients but is associated with a high rate of hospital infections. Inhalation mucolytic therapy has emerged as a feasible, easy-to-use, and economical therapeutic option with potentially beneficial effects for reduction of in-hospital infections. Use of mucolytic inhalation therapy can potentially decrease the incidence of VAP and consumption of reserve group antibiotics, shorten the length of stay at the intensive care unit (ICU), and reduce healthcare expenses. Existing literature does not report clinically significant adverse reactions to the inhalation therapy utilized in this study.

### 2.2. Ethical and Institutional Considerations

All the proceedings and clinical research were conducted by following the ethical standards and amendments of the Declaration of Helsinki. The study protocol was approved by the Ethical Committee of the University Hospital of Split, Croatia (no. 2181-147-01/06/M.S.-20-02). Access to the project documentation and patient identity was allowed only to the principal investigators. The collected data were used exclusively for this research. Relevant data were processed electronically, and principal investigators adhered to a protocol for data protection.

### 2.3. Informed Consent

Enrolled patients were unable to give informed consent for study participation due to an impaired level of consciousness during mechanical ventilation, but it was gathered from their relatives/family members according to the local protocols. This was approved by the relevant Ethical Committee of the University Hospital of Split (no. 2181-147-01/06/M.S.-20-02).

### 2.4. Study Design and Patients

This single-center, randomized controlled trial was conducted at the ICU of the Clinic of Anaesthesiology and Intensive Care of the University Hospital of Split. A total of 175 eligible adult patients with COVID-19 who were treated with mechanical ventilation due to respiratory insufficiency between October 2020 and June 2021 were enrolled. All enrolled patients suffered from severe ARDS as defined by the Berlin criteria (PaO_2_/FiO_2_ is ≤100 mmHg on ventilator settings that include PEEP ≥ 5 cm H_2_O) [10]. Active COVID-19 was diagnosed and confirmed in all patients with the specific 3-sequence (E-gen; ORF 1ab-gen; N-gen) reverse transcription-polymerase chain reaction (RT-PCR) test (Liferiver Novel Coronavirus RT-PCR Kit/MIC qPCR cycler).

Patients were randomized and allocated to the control group (no routine inhalation) or one of the treatment arms (inhalation of N-acetylcysteine; hypertonic 5% saline solution; or 8.4% sodium bicarbonate). All inhalation therapies were given twice daily at 12 h intervals. The first inhalation was applied within 12 h of the patient’s admission to the ICU. There are no recommendations for routine prophylactic use of mucolytic inhalation therapy in mechanically ventilated patients (no “gold standard”), so we did not apply any routine inhalation therapy to patients in the control group. However, similar to existing studies, it was not forbidden to administer non-routine, nebulized solutions on a case-to-case basis, per physician’s discretion, if there was a strong indication (severe thick and tenacious secretions or bronchospasm) [11].

Exclusion criteria were: recent polytrauma; pregnancy; severe hemodynamic instability defined as a need for vasopressor/inotropic therapy or mechanical circulatory support; cardiogenic pulmonary edema or edema due to fluid overload; length of stay at the ICU less than 3 days; and verified bacterial infection prior to ICU admission.

This study was registered at the web-based resource www.clinicaltrials.gov (accessed on 7 April 2022, Bethesda, MD, USA) with the registration number (NCT 04755972; Supplementary File S1). The flow diagram of the study is shown in Supplementary Figure S1. The Consolidated Standards of Reporting Trials (CONSORT) checklist for randomized controlled trials is reported in Supplementary File S2. There was no external funding for this study.

### 2.5. Data Sources

Relevant medical data were obtained from the hospital’s electronic medical records, therapeutic lists, and paper documentation.

### 2.6. Randomization Process

Patients were randomized using an online platform for randomization www.random.org (accessed on 7 April 2022, Randomness and Integrity Services Ltd., Dublin, Ireland). Patients were allocated on a 1:1 basis by random assignment to a control group or one of the treatment arms.

### 2.7. Research Outcomes

The primary outcome of this study was the incidence of VAP. The secondary outcome was the all-cause mortality within a 28-day time period. VAP was defined as a microbiologically confirmed respiratory isolate accompanied by one of the following: novel lung infiltrate on chest radiograph; new-onset fever in the ICU (>38.3 °C); new-onset neutrophilia (>85% or left shift); purulent tracheobronchial secretions. Radiologic assessment was performed by an experienced radiologist with particular focus on COVID-19-related pathology. The radiologist was blinded in his interpretation of the chest radiographs.

In the Croatian health system, patients who receive mechanical ventilation treatment for longer than 4 days have equal healthcare costs (~16,000 €), so the authors were unable to investigate the economic aspects of the investigated therapy.

### 2.8. Clinical and Laboratory Assessment

Detailed patient characteristics were gathered, including anthropometric data (age, sex), comorbidities, laboratory parameters (complete blood count, C-reactive protein (CRP), blood urea nitrogen (BUN), creatinine, LDH, high-sensitivity troponin T (hsTnT), NT-proBNP, liver enzymes, coagulation parameters, electrolytes, albumin, glucose, arterial blood gas analysis), and hospitalization-related factors (length of stay, duration of mechanical ventilation). The estimated glomerular filtration rate (eGFR) was calculated using a CKD-EPI formula (17). Comorbidity burden was estimated using the Charlson comorbidity index (CCI), as previously described (18).

### 2.9. Microbiological Analysis

Microbiological analysis was conducted following the medical standards. The samples for microbiological analysis were carefully aspirated with a sterile catheter through the endotracheal tubes and were then properly stored in a sterile container. Strict measures were applied to prevent the contamination of the samples. Sampling was performed by an experienced medical nurse/technician from the ICU team. Microbiological analysis of the endotracheal aspirate was conducted by an experienced medical doctor specialized in medical microbiology at the Department of Microbiology of the University Hospital of Split. Microbiological sampling was performed routinely every 5 days or additionally in the case of clinical indication, per physician’s discretion.

Endotracheal aspirates were cultured on blood agar and CHROMagar Orientation medium (CHROMagar, Paris, France). The plates were checked for bacterial growth after 24–48 h, and quantitative cultures were interpreted using a threshold of >10^5^ colony-forming units (CFU)/mL to designate the clinical significance. The final identification of bacterial and fungal species was performed using MALDI-TOF MS (Bruker biotyper).

The following bacteria were finally isolated in this study: methicillin-resistant *Staphylococcus aureus* (MRSA); *Streptococcus pneumoniae*; *Corynebacterium* species; *Acinetobacter* species; *Pseudomonas* species; *Klebsiella* species; *Enterobacter* species; *Proteus* species; *Stenotrophomonas* species; *Morganella* species; *Elizabethkingia* species; *Escherichia coli*; *Achromobacter xylosoxidans*; and *Serratia* species. Bacteria that are able to grow in the absence of oxygen were considered anaerobic, including facultative and obligate anaerobic bacteria. The following bacteria were considered anaerobic: *Klebsiella* species; *Streptococcus pneumoniae*; *Enterobacter* species; *Proteus* species; *Morganella* species; *Escherichia coli*; *Serratia* species. Isolates that showed resistance to ≥2 antibiotics during antibiotic susceptibility testing were considered multi-drug resistant (MDR) bacteria. MRSA phenotype interpretation was performed using EUCAST clinical breakpoints, while the MRSA phenotype was detected according to cefoxitine resistance in *Staphylococcus aureus*. Both reference strains of *Staphylococcus aureus* ATCC 29213 and *Staphylococcus aureus* NCTC 12493 (mec A-positive) were used for quality control.

The following fungi were finally isolated in this study: *Candida albicans*; *Candida glabrata*; *Candida parapsilosis*; and *Aspergillus* species.

### 2.10. Interventions

#### 2.10.1. 5% Saline (Hypertonic NaCl Solution)

The 5% saline represents hypertonic NaCl solution, and its safety has been consistently demonstrated in different studies. Several beneficial effects of this solution include osmotic gradient on the surface of the bronchi [12,13,14], reduced viscosity [15,16], anti-inflammatory activity [15,16], and possible antimicrobial activity [17,18]. Most adverse events are mild and resolved spontaneously, such as cough, dyspnea, throat irritation, and salty taste after inhalation [19]. Everard et al. reported only one serious adverse event (bradycardia and desaturation) during 5% saline application, which spontaneously resolved in the following hours [20].

#### 2.10.2. N-Acetylcysteine Inhalation

Nebulized N-acetylcysteine is one of the most widely used medications for reducing sputum viscosity and stimulating expectoration [8]. The safety of N-acetylcysteine solution has been previously demonstrated. N-acetylcysteine can cause intermittent, localized irritation, mild bronchospasm, a burning sensation in the nasal mucosa, rhinorrhea, nausea, and vomiting [21]. However, these potential side effects are not largely relevant for the study population due to active sedation and mechanical ventilation.

#### 2.10.3. 8.4% Bicarbonate Inhalation

Bicarbonate solution is important for mucus formation and its viscosity [22]. Several research works studied the inhalation application of bicarbonate solution and have demonstrated the feasibility of this treatment. It was used on patients with cystic fibrosis [7], the healthy non-smoker population, and mechanically ventilated COVID-19 patients [23]. No adverse events associated with the use of 8.4% bicarbonate inhalation were reported in the available literature [7].

### 2.11. Statistical Analysis

Statistical analysis was conducted according to standard statistical methods. Categorical variables were expressed as numbers (percentages) and analyzed using the chi-squared test, while continuous data were expressed as median (interquartile range (IQR)) and analyzed using the Kruskal–Wallis test. To assess the predictors of any bacterial or fungal infection, univariate and multivariate binomial logistic regression analyses were conducted and described as the odds ratio (OR) and adjusted odds ratios (aOR), respectively, with 95% confidence intervals (95% CI). A two-sided *p*-value of <0.05 was considered significant. To account for type 1 errors with multiple comparisons, the Bonferroni correction was used for secondary exploratory findings and is denoted under the corresponding table where appropriate. Statistical data analysis was carried out using the Statistical Package for the Social Sciences (SPSS) software (IBM Corp, New York, NY, USA; version 20).

## 3. Results

### 3.1. Baseline Characteristics

There was no statistically significant difference in the baseline patient characteristics between the study groups, except in the proportion of female patients, which was the highest in the 5% saline group (42.9% vs. 16.7–23.1% in other groups, *p* = 0.047), the prevalence of prior smoking, which was the highest in 8.4% bicarbonate group (52.0% vs. 8.3–31.4% in other groups, *p* = 0.012), and the prevalence of prior percutaneous coronary intervention, which was the highest in the 5% saline group (11.9% vs. 0.0–11.9% in other groups, *p* = 0.048) (Table 1). When looking at the laboratory parameters, there was no statistically significant difference between the study groups (Supplementary Table S1).

### 3.2. Incidence of Bacterial Pneumonia

**Figure 1:**
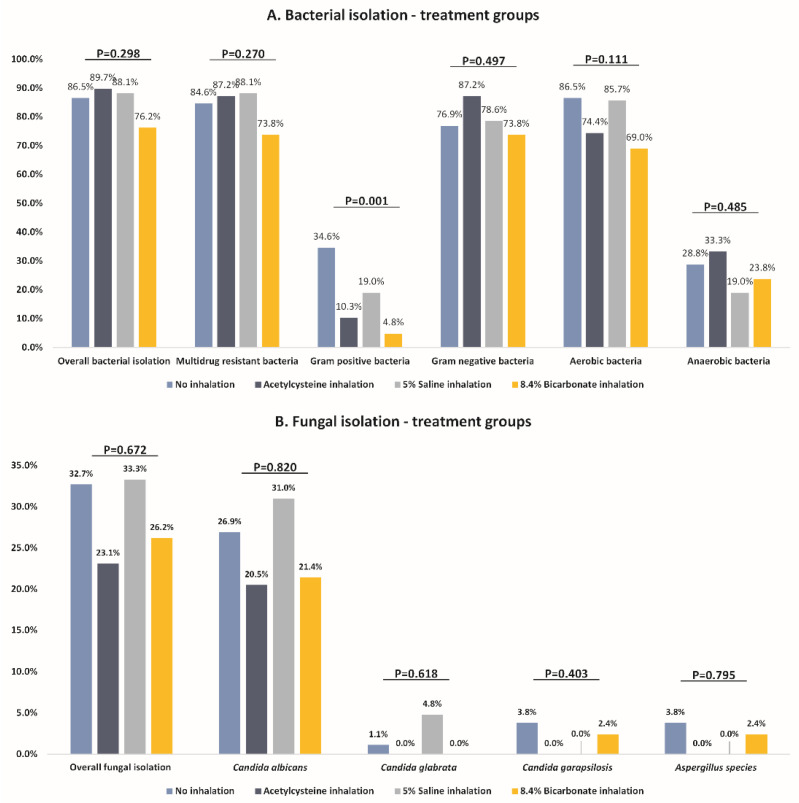
Comparison of different microbiological isolation between the study groups: (**A**) bacterial isolation; (**B**) fungal isolation.

When comparing the incidence of bacterial pneumonia across the study groups, there was no difference in the incidence of any bacterial pneumonia (86.5% in the control group vs. 89.7% in the N-acetylcysteine inhalation group vs. 88.1% in the 5% saline group vs. 76.2% in the bicarbonate inhalation group, *p* = 0.298), MDR bacterial pneumonia (84.6% in the control group vs. 87.2% in the N-acetylcysteine inhalation group vs. 88.1% in the 5% saline group vs. 73.8% in the bicarbonate inhalation group, *p* = 0.270), Gram-negative bacterial pneumonia (76.9% in the control group vs. 87.2% in the N-acetylcysteine inhalation group vs. 78.6% in the 5% saline group vs. 73.8% in the bicarbonate inhalation group, *p* = 0.497), aerobic bacterial pneumonia (86.5% in the control group vs. 74.4% in the N-acetylcysteine inhalation group vs. 85.7% in the 5% saline group vs. 69.0% in the bicarbonate inhalation group, *p* = 0.111), and anaerobic bacterial pneumonia (28.8% in the control group vs. 33.3% in the N-acetylcysteine inhalation group vs. 19.0% in the 5% saline group vs. 23.8% in the bicarbonate inhalation group, *p* = 0.485). However, the control group had a statistically significantly higher incidence of Gram-positive bacterial pneumonia (34.6%), followed by the 5% saline group (19.0%), N-acetylcysteine group (10.3%), and bicarbonate group (4.8%; *p* = 0.001) (Table 2 and Figure 1A).

**Figure 2:**
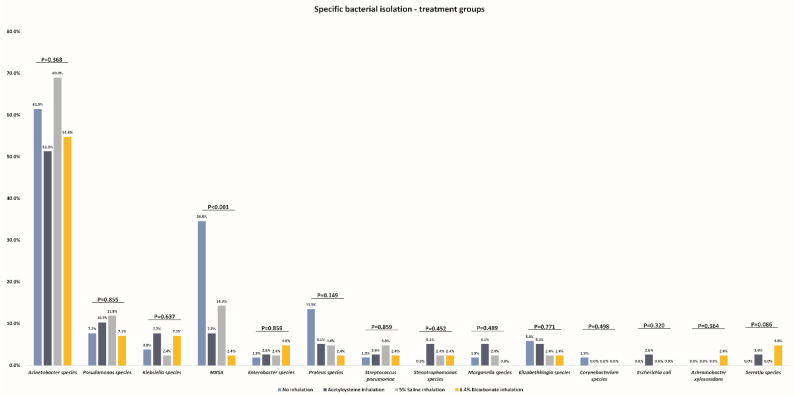
Comparison of specific bacterial isolation between the study groups.

When looking at the specific bacteria isolated, the difference in MRSA incidence was the major driver of between-group differences with a statistically significantly higher incidence in the control group (34.6%), followed by the 5% saline group (14.3%), N-acetylcysteine group (7.7%), and bicarbonate group (2.4%; *p* < 0.001). There was no significant between-group difference in isolation of any other specific bacteria (Table 2 and Figure 2).

### 3.3. Incidence of Fungal Pneumonia

When comparing the incidence of fungal pneumonia across the study groups, there was no difference in the incidence of any fungal pneumonia (32.7% in the control group vs. 23.1% in the N-acetylcysteine inhalation group vs. 33.3% in the 5% saline group vs. 26.2% in the bicarbonate inhalation group, *p* = 0.672). This pattern persisted when evaluating specific fungal types (*p* > 0.05) (Table 3 and Figure 1B).

### 3.4. Predictors of VAP

When evaluating the predictors of bacterial VAP, longer duration of ventilator therapy was significantly associated with any bacterial pneumonia in both univariate (OR 1.20, 95% CI 1.09–1.32, *p* < 0.001) and multivariate analysis (aOR 1.14, 95% CI 1.01–1.29, *p* = 0.038). Longer duration of hospitalization was significantly associated with any bacterial pneumonia only in univariate analysis (OR 1.07, 95% CI 1.02–1.12, *p* = 0.004), while there was no significant association between age, sex, Charlson comorbidity index, prior smoking, albumin, or glucose and bacterial VAP (Table 4).

Similarly, longer duration of ventilator therapy was significantly associated with any fungal VAP in both univariate (OR 1.04, 95% CI 1.01–1.07, *p* = 0.015) and multivariate analysis (aOR 1.05, 95% CI 1.01–1.10, *p* = 0.028), while there was no significant association between age, sex, longer duration of hospitalization, Charlson comorbidity index, prior smoking, albumin, or glucose and fungal VAP (Supplementary Table S2).

### 3.5. Comparison of Mortality

**Figure 3:**
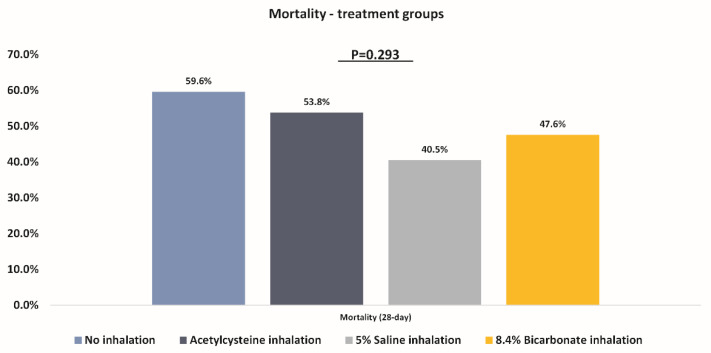
Comparison of mortality between the study groups.

There was no statistically significant difference in 28-day all-cause mortality between the study groups (Table 3 and Figure 3).

### 3.6. Adverse Events

There were no significant adverse events related to the utilization of inhalation therapies, except for bronchospasm, which developed in a patient from the N-acetylcysteine treatment arm (N = 1, 2.4%) and a patient from the 8.4% bicarbonate treatment arm (N = 1, 2.6%). Both events had mild clinical course, and the patients fully recovered spontaneously or shortly after application of bronchodilator inhalation therapy.

### 3.7. Power Analysis

Post hoc power size analysis for a specified sample size was calculated using the study data (differences in the incidence of any bacterial pneumonia) by controlling for type 1 error at alpha 0.05. The results indicate that the study was not powered to detect a significant difference in the incidence of any bacterial pneumonia across the study groups with a power of 44.5%.

## 4. Discussion

This randomized controlled trial evaluated the effects of inhalation therapy on the incidence of bacterial and fungal VAP in mechanically ventilated COVID-19 patients. It is the first study to systematically investigate all the aforementioned inhalation agents in COVID-19 ICU settings. There are several important results of this trial. First, mechanically ventilated patients with COVID-19 have high incidence of bacterial and fungal VAP. Second, the incidence of VAP was strongly and independently associated with the duration of mechanical ventilation. Third, inhalation therapy had no effect on the overall incidence of bacterial or fungal VAP. Yet, exploratory analyses suggest potential, beneficial effects of inhalation therapy on the incidence of Gram-positive bacterial VAP. These results were particularly driven by reduced incidence of MRSA-caused pneumonia, while the strongest effect was observed with 8.4% bicarbonate inhalation. Therefore, inhalation therapy does not yield any beneficial effect on the overall VAP incidence, but further, adequately powered studies are deemed necessary to explore any effects on Gram-positive bacterial and other specific VAP. This therapy is a feasible, easy-to-use, and economical treatment option during the mechanical ventilation of COVID-19 patients.

VAP represents a devastating complication in mechanically ventilated patients. As well as requiring delicate management, its diagnosis in patients with ARDS is usually challenging. Difficult differentiation of novel, VAP-associated pulmonary infiltrations from other causes, such as ARDS progression, pulmonary edema, or atelectasis, represents a clinical challenge. Furthermore, differentiation of tracheobronchitis and pneumonia also represents an immense task. Finally, corticosteroid therapy suppresses immunologic response and blunts the increase in laboratory parameters, aggravating the diagnosis [5]. Irrevocably, VAP represents a substantial, global health problem with high in-hospital mortality and a substantial, economic healthcare burden [11].

Previous studies showed an emerging incidence of VAP in COVID-19 settings, ranging from 48% to 79% [11,24,25,26]. The incidence of VAP in COVID-19 patients seems to be even higher compared to in non-COVID-19 patients treated in ICU [3]. Several pathophysiological mechanisms may mediate these findings in COVID-19 patients, including profound hypoxemia and lung damage [3], prolonged duration of mechanical ventilation and prone positioning [3,27], higher incidence of pulmonary embolism/infarction, and superimposed bacterial infection [28,29]. The use of immunomodulatory therapy, such as high-dose corticosteroids or interleukin antagonists (e.g., tocilizumab), can also suppress immune response and precipitate the development of VAP in COVID-19 patients [30,31]. This study detected even higher incidence of bacterial VAP across all study groups, ranging from 76% to 90%. One of the possible causes for such high incidence of VAP in this study could be more frequent sampling for study purposes and increased diagnostic awareness. However, this could also contribute to other novel aspects of the study, including the evaluation of the natural course of ICU-acquired pneumonia, as well as the evaluation of risk factors for VAP.

Viral infections suppress the host immunity and facilitate the entry of bacterial pathogens [32], mediated by morpho-functional airway impairment such as induced cell apoptosis, hyperplasia, decreased cellular respiration, and impaired surfactant function [33,34]. However, one of the most important factors for VAP development is the duration of mechanical ventilation [3,28,30,35]. The association of prolonged mechanical ventilation with both bacterial and fungal VAP was confirmed in this study. The longer the time spent on invasive ventilation, the more opportunities microorganisms have to colonize, leading to greater utilization of broad-spectrum antibiotics and increased fungal emergence. Critically ill patients are also more likely to receive invasive management with vascular or urinary catheters, resulting in increased susceptibility to bacterial superinfection with MDR pathogens [36]. This should encourage intensive care physicians to routinely re-assess the need for mechanical ventilation and strive to wean patients off mechanical ventilation.

However, certain patients are fully dependent on mechanical ventilation, and inhalation therapy represents a promising therapeutic option to reduce VAP in this population. Hypertonic 5% saline solution has several pharmacodynamic effects on the surface of bronchi, including osmotic gradient [12,13,14] and reduced viscosity [15,16]. Some authors even speculated it could have anti-inflammatory activity [15,16] or antimicrobial activity by inhibiting growth of *Pseudomonas aeruginosa* [17,18]. The Cochrane review on the use of 5% saline solution suggested potential benefits in older children and adults with cystic fibrosis [9]. A similar Cochrane review suggested beneficial effects of nebulized hypertonic saline in infants with acute bronchiolitis [37].

Other inhalation agents also showed promising mucolytic properties. N-acetylcysteine reduced the bacterial formation of biofilm among intubated patients in a study by Qu et al. [38]. However, a large NEBULAE study did not detect any effect of routine preventive use of N-acetylcysteine (in combination with salbutamol) on VAP incidence [39]. Yet, the limitations of that study call for caution due to high sample heterogeneity, i.e., the study included all patients on mechanical ventilation for more than 24 h, but only a few of them had ARDS according to the international criteria [39]. Bicarbonate inhalation was associated with significant clinical improvement in COVID-19 patients with ARDS, as reported in a case series by Wardeh et al. [23]. A literature review by Icard and Rubio did not find any report of significant adverse events of bicarbonate inhalation [6]. Bicarbonate inhalation proved to be safe and well tolerated in patients with cystic fibrosis and obstructive pulmonary diseases [7]. In vitro and in vivo animal studies showed that bicarbonate reduces the viscosity of sputum [6,22,40,41], possibly by expanding mucin granules [6]. Other possible mechanisms of action of bicarbonate inhalation may include a local increase in pH, creating an unfavourable environment for the growth of bacteria such as *Acinetobacter* spp.

Based on our findings and the existing literature, there is currently insufficient evidence to support the routine use of preventive inhalation therapy with N-acetylcysteine, 5% saline, or 8.4% bicarbonate to decrease the incidence of VAP in mechanically ventilated COVID-19 patients. However, the evidence from this trial, although not adequately powered, suggests potential beneficial effects of inhalation therapy on the incidence of Gram-positive bacterial VAP, a finding which should be confirmed in further studies. Although the most common causes of VAP are Gram-negative bacteria, MRSA causes approximately 7% of VAP [42,43,44]. The present study found even higher incidence of MRSA VAP in the control group (up to 35%), highlighting the importance of this pathogen in critically ill COVID-19 patients. Any efforts to decrease this complication are warranted. All three inhalational treatments have a good safety profile and are inexpensive, with the highest cost being for nebulized N-acetylcysteine (a price per dose of approximately EUR 2.4). With respect to the possible beneficial effects, good safety profile, and low cost, any of the studied treatments could be considered for VAP prevention in mechanically ventilated COVID-19 patients, per physician’s discretion. This could be even more important in ICUs with high incidence of Gram-positive VAP. However, lack of high-quality data on this topic should be emphasized, warranting further studies.

Multivariable adjustment was not conducted in this study due to well-balanced baseline characteristics across the study groups. In addition, it was not necessary to adjust for the baseline immunosuppressive or antibiotic treatment because all enrolled patients received a standard dose of intravenous dexamethasone (8 mg) during the first 10 days, along with the same broad-spectrum, empirical antibiotic, according to local ICU protocol, until microbiological isolates were obtained.

This study had several limitations. Limited sample size could have decreased the statistical power of the study and detection of between-group difference in less prevalent infections. The study was not powered to draw conclusions on the secondary findings; these were presented only as exploratory, hypothesis-generating findings. Furthermore, due to routine microbiological sampling and the limitations of VAP diagnostic criteria, it is possible that the incidence rates were increased by capturing subclinical infections. Clinical relevance of particular, isolated bacteria, such as the *Corynebacterium* species, which is commonly considered as a contaminant in respiratory specimens, is questionable. In order to account for this, cultures were quantitatively interpreted using a threshold of >10^5^ CFU/mL to designate the clinical significance. Furthermore, the diagnosis of VAP is challenging in ARDS patients, and microbiological confirmation should not reduce the diagnostic specificity.

In addition, selection bias could not be fully eliminated with this study due to the specific characteristics of the population. Additionally, the potential impact of unmeasured variables on the outcomes could not be fully removed. Although the authors used the Bonferroni correction for secondary analyses, it was not possible to fully eliminate the possibility of type 1 errors. Finally, this trial represents a negative clinical trial as there was no difference made by the intervention on the primary study outcome, but it can still provide clinically relevant findings.

## 5. Conclusions

In conclusion, preventive inhalation therapy with N-acetylcysteine, 5% saline, or 8.4% bicarbonate had no effect on the overall VAP incidence or mortality in mechanically ventilated COVID-19 patients. Further studies should explore the secondary findings of this study such as the reduction of Gram-positive and MRSA-caused VAP in patients undergoing inhalation treatment.

## Figures and Tables

**Table 1:** Comparison of laboratory parameters between the study groups.

Variables	Inhalation Type				*p*-Value
	Control Group—No Routine Inhalation (n = 52; 29.7%)	N-Acetylcysteine Inhalation (n = 39; 22.3%)	5% Saline Inhalation (n = 42; 24.0%)	8.4% Sodium Bicarbonate Inhalation (n = 42; 24.0%)	
**Age (years)**	68.0 (62.0–74.5)	68.5 (60.0–73.0)	63.0 (53.3–68.8)	63.5 (57.8–71.3)	0.134 †
**Female sex**	12 (23.1%)	11 (28.2%)	18 (42.9%)	7 (16.7%)	0.047 *
**Systolic blood pressure (mmHg)**	124.0 (112.5–147.0)	129.5 (117.5–145.0)	140.0 (126.3–157.5)	140.0 (133.5–151.5)	0.773 †
**Diastolic blood pressure (mmHg)**	71.0 (65.0–80.0)	72.5 (70.0–80.5)	80.0 (70.8–88.8)	76.0 (73.8–90.0)	0.934 †
**Heart rate (/min)**	97.0 (80.0–110.5)	80.0 (76.0–91.8)	87.5 (80.5–93.8)	89.0 (77.0–104.3)	0.388 †
**Disease duration upon intubation (days)**	12.0 (9.0–17.0)	11.0 (9.8–14.3)	10.5 (9.0–14.0)	11.0 (8.8–15.0)	0.479 †
**Active smoking**	1 (2.3%)	3 (9.7%)	3 (7.9%)	1 (2.9%)	0.425 *
**Prior smoking**	11 (31.4%)	2 (8.3%)	9 (30.0%)	13 (52.0%)	0.012 *
**Arterial hypertension**	32 (64.0%)	24 (61.5%)	25 (59.5%)	22 (53.7%)	0.787 *
**Diabetes mellitus**	19 (38.0%)	6 (15.4%)	10 (23.8%)	12 (29.3%)	0.111 *
**Dyslipidaemia**	10 (20.0%)	5 (12.8%)	5 (11.9%)	8 (20.0%)	0.607 *
**Chronic heart failure**	5 (10.0%)	2 (5.1%)	2 (4.8%)	0 (0.0%)	0.206 *
**Atrial fibrillation**	7 (14.0%)	5 (12.8%)	5 (11.9%)	5 (12.2%)	0.991 *
**Prior acute myocardial infarction**	3 (6.0%)	2 (5.1%)	6 (14.3%)	4 (9.8%)	0.426 *
**Prior percutaneous coronary intervention**	0 (0.0%)	2 (5.1%)	5 (11.9%)	1 (2.4%)	0.048 *
**Prior coronary artery bypass grafting**	2 (4.0%)	1 (2.6%)	2 (4.8%)	3 (7.3%)	0.780 *
**Prior cerebrovascular incident**	2 (4.1%)	2 (5.1%)	0 (0.0%)	3 (7.3%)	0.394 *
**Peripheral artery disease**	1 (2.0%)	1 (2.6%)	1 (2.4%)	3 (7.3%)	0.500 *
**COPD/asthma**	9 (18.0%)	1 (2.6%)	4 (9.5%)	5 (12.2%)	0.140 *
**Charlson comorbidity index**	4.0 (2.0–5.0)	3.0 (2.0–3.3)	2.0 (2.0–3.8)	3.0 (2.0–4.0)	0.147 †
**Deep vein thrombosis**	0 (0.0%)	0 (0.0%)	0 (0.0%)	0 (0.0%)	/
**Pulmonary embolism**	2 (3.9%)	1 (2.6%)	2 (4.8%)	0 (0.0%)	0.574 *
**Pleural effusion**	22 (42.3%)	12 (30.8%)	17 (40.5%)	17 (40.5%)	0.699 *
**Duration of ventilator therapy (days)**	13.0 (8.0–21.0)	8.5 (6.0–18.3)	12.5 (5.0–19.0)	10.5 (5.0–22.0)	0.364 †
**Length of stay (days)**	25.0 (17.5–34.0)	25.0 (16.8–35.0)	25.0 (18.0–31.8)	30.5 (19.8–54.5)	0.949 †

Data are expressed as number (percent) or median (interquartile range). * Chi-square test; † Kruskal–Wallis test. Abbreviations: COPD—chronic obstructive pulmonary disease.

**Table 2:** Comparison of bacterial isolation between the study groups.

Variables	Inhalation Type				*p*-Value *
	Control Group—No Routine Inhalation (n = 52; 29.7%)	N-Acetylcysteine Inhalation (n = 39; 22.3%)	5% Saline Inhalation (n = 42; 24.0%)	8.4% Sodium Bicarbonate Inhalation (n = 42; 24.0%)	
**Any bacterial pneumonia**	45 (86.5%)	35 (89.7%)	37 (88.1%)	32 (76.2%)	0.298
**MDR isolation**	44 (84.6%)	34 (87.2%)	37 (88.1%)	31 (73.8%)	0.270
**Gram-positive bacteria**	18 (34.6%)	4 (10.3%)	8 (19.0%)	2 (4.8%)	0.001 †
**Gram-negative bacteria**	40 (76.9%)	34 (87.2%)	33 (78.6%)	31 (73.8%)	0.497
**Aerobic bacteria**	45 (86.5%)	29 (74.4%)	36 (85.7%)	29 (69.0%)	0.111
**Anaerobic bacteria**	15 (28.8%)	13 (33.3%)	8 (19.0%)	10 (23.8%)	0.485
**Specific bacteria type:**					
***Acinetobacter*** **species**	32 (61.5%)	20 (51.3%)	29 (69.0%)	23 (54.8%)	0.368
***Pseudomonas*** **species**	4 (7.7%)	4 (10.3%)	5 (11.9%)	3 (7.1%)	0.855
***Klebsiella*** **species**	2 (3.8%)	3 (7.7%)	1 (2.4%)	3 (7.1%)	0.637
**MRSA**	18 (34.6%)	3 (7.7%)	6 (14.3%)	1 (2.4%)	<0.001 ‡
***Enterobacter*** **species**	1 (1.9%)	1 (2.6%)	1 (2.4%)	2 (4.8%)	0.859
***Proteus*** **species**	7 (13.5%)	2 (5.1%)	2 (4.8%)	1 (2.4%)	0.149
***Streptococcus pneumoniae***	1 (1.9%)	1 (2.6%)	2 (4.8%)	1 (2.4%)	0.859
***Stenotrophomonas*** **species**	0 (0.0%)	2 (5.1%)	1 (2.4%)	1 (2.4%)	0.452
***Morganella*** **species**	1 (1.9%)	2 (5.1%)	1 (2.4%)	0 (0.0%)	0.489
***Elizabethkingia*** **species**	3 (5.8%)	2 (5.1%)	1 (2.4%)	1 (2.4%)	0.771
***Corynebacterium*** **species**	1 (1.9%)	0 (0.0%)	0 (0.0%)	0 (0.0%)	0.498
***Escherichia coli***	0 (0.0%)	1 (2.6%)	0 (0.0%)	0 (0.0%)	0.320
***Achromobacter xylosoxidans***	0 (0.0%)	0 (0.0%)	0 (0.0%)	1 (2.4%)	0.364
***Serratia*** **species**	0 (0.0%)	1 (2.6%)	0 (0.0%)	2 (4.8%)	0.249

Data are expressed as number (percent). * Chi-square test. † Significant after Bonferroni correction (*p* < 0.01). ‡ Significant after Bonferroni correction (*p* < 0.003). Abbreviations: MDR—multi-drug resistant bacteria, MRSA—methicillin-resistant *Staphylococcus aureus*.

**Table 3:** Comparison of fungal isolation and all-cause mortality between the study groups.

Variables	Inhalation Type				*p*-Value *
	Control Group—No Routine Inhalation (n = 52; 29.7%)	N-Acetylcysteine Inhalation (n = 39; 22.3%)	5% Saline Inhalation (n = 42; 24.0%)	8.4% Sodium Bicarbonate Inhalation (n = 42; 24.0%)	
**Fungal isolation**	17 (32.7%)	9 (23.1%)	14 (33.3%)	11 (26.2%)	0.672
**Specific fungal type**					
***Candida albicans***	13 (26.9%)	8 (20.5%)	12 (31.0%)	9 (21.4%)	0.820
***Candida glabrata***	1 (1.9%)	0 (0.0%)	1 (4.8%)	0 (0.0%)	0.618
***Candida parapsilosis***	2 (3.8%)	0 (0.0%)	0 (0.0%)	1 (2.4%)	0.403
***Aspergillus* species**	1 (3.8%)	0 (0.0%)	0 (0.0%)	1 (2.4%)	0.795
**All-cause mortality (28-day)**	31 (59.6%)	21 (53.8%)	17 (40.5%)	20 (47.6%)	0.293

Data are expressed as numbers (percent). * Chi-square test. Abbreviations: none.

**Table 4:** Predictors of any bacteria isolation.

Variables	Univariate Analysis		Multivariate Analysis	
	OR (95% CI)	*p*-Value	aOR (95% CI)	*p*-Value
**Age**	1.01 (0.98–1.06)	0.484	1.02 (0.96–1.08)	0.617
**Female sex**	0.45 (0.19–1.07)	0.070	0.30 (0.07–1.33)	0.113
**Duration of ventilator therapy (days)**	1.20 (1.09–1.32)	<0.001	1.14 (1.01–1.29)	0.038
**Hospitalization duration (days)**	1.07 (1.02–1.12)	0.004	1.03 (0.98–1.08)	0.191
**Charlson comorbidity index**	1.01 (0.78–1.29)	0.968	0.92 (0.58–1.46)	0.724
**Prior smoking**	0.87 (0.30–2.53)	0.792	0.60 (0.13–2.84)	0.520
**Albumin (g/L)**	0.99 (0.88–1.12)	0.917	0.94 (0.80–1.10)	0.408
**Glucose (mmol/L)**	0.98 (0.91–1.06)	0.616	0.93 (0.83–1.04)	0.220

Abbreviations: OR—odds ratios; aOR—adjusted odds ratios.

## Data Availability

Data are available upon reasonable request to the corresponding author.

## Supplementary Materials

The following supporting information can be downloaded at: https://www.mdpi.com/article/10.3390/microorganisms10061118/s1. Table S1. Comparison of laboratory parameters between the study groups; Table S2. Predictors of any fungal isolation; Figure S1. Flow diagram. File S1. ClinicalTrials Confirmation; File S2. CONSORT Checklist.
