# Supplementary material for: Effects of Different Inhalation Therapy on Ventilator-Associated Pneumonia in Ventilated COVID-19 Patients: A Randomized Controlled Trial
Source: Microorganisms. 2022 May 28;10(6):1118. doi: 10.3390/microorganisms10061118 (PMC9228146; doi:10.3390/microorganisms10061118)
Supplement: Supplementary file 1 [file microorganisms-10-01118-s001.zip › microorganisms-1662466-supplementary/Supplementary File S1_ClinicalTrials Confirmation.pdf]

ClinicalTrials.gov Protocol Registration and Results System (PRS) Receipt
Release Date: February 11, 2022

ClinicalTrials.gov ID: NCT04755972

Study Identification

Unique Protocol ID: 2181-147-01/06/M.S.-20-02

Brief Title: Mucolytics in Patients on Invasive Mechanical Ventilation Due to Severe Acute Respiratory Syndrome Coronavirus 2

Official Title: Mucolytic Agents and Ventilator-associated Pneumonia in Patients on Invasive Mechanical Ventilation Due to Severe Acute Respiratory Syndrome Coronavirus 2

Secondary IDs:

Study Status

Record Verification: February 2022

Overall Status: Completed

Study Start: October 1, 2020 [Actual]

Primary Completion: July 1, 2021 [Actual]

Study Completion: July 29, 2021 [Actual]

Sponsor/Collaborators

Sponsor: Clinical Hospital Center, Split

Responsible Party: Principal Investigator
Investigator: Nikola Delić [ndelic]
Official Title: MD, intensive care specialist
Affiliation: Clinical Hospital Center, Split

Collaborators:

Oversight

U.S. FDA-regulated Drug: No

U.S. FDA-regulated Device: No

U.S. FDA IND/IDE: No

Human Subjects Review: Board Status: Approved
Approval Number: 500-03/21-01/04
Board Name: Ethics Committee
Board Affiliation: Clinical Hospital Center Split
Phone: +38521556111
Email: eticko.povjerenstvo@kbsplit.hr
Address:

Spinciceva 1, 21000 Split, Croatia

Data Monitoring:

Study Description

Brief Summary: It is planned to include patients over 18 years of age of both sexes, admitted to the Intensive Care Unit of Clinical Hospital Centre Split for respiratory insufficiency caused by severe acute respiratory syndrome coronavirus 2 in need of invasive mechanical ventilation.

The patients will be divided into four groups. Group 1 will receive N-acetylcysteine inhalation, Group 2 will receive inhalation with a 5% sodium chloride solution, and Group 3 will receive inhalation of 8.4% sodium bicarbonate, group 4 is a control group and will not routinely receive inhaled mucolytics preventively.

All inhalations will be given twice a day 12 hours apart. The first inhalation will be included within 12 hours of the patient being enrolled in the Intensive Care Unit. Patients will be randomized according to the type of inhalation they will receive, randomization will be done by all researchers through the random.org website, and the inhalation will be given by a nurse according to the agreed protocol.

RESEARCH GOALS

The aim of this study is to determine whether there is a difference in the frequency and duration of ventilator-associated pneumonia (VAP) and whether there is a difference in the number of days spent on mechanical ventilation and in mortality in these four groups of patients.

Hypothesis

Coronavirus disease 2019 patients on invasive mechanical ventilation and preventive sodium bicarbonate inhalation will have a lower incidence of ventilator-associated pneumonia and fewer days spent on invasive mechanical ventilation than patients inhaled with N-acetylcysteine, 5% saline, or patients without preventive inhalation.

Detailed Description:

Conditions

Conditions: Corona Virus Infection
Pneumonia, Ventilator-Associated

Keywords: coronavirus disease 2019
Pneumonia, Ventilator-Associated
hypertonic saline
bicarbonate
N-acetylcysteine

Study Design

Study Type: Interventional

Primary Purpose: Prevention

Study Phase: N/A

Interventional Study Model:	Parallel Assignment
Number of Arms:	4
Masking:	None (Open Label)
Allocation:	Randomized
Enrollment:	175 [Actual]

Arms and Interventions

Arms	Assigned Interventions
Active Comparator: N-acetylcysteine<br>Inhalation of 5 ml-s of N-acetylcysteine every 12 hours.	Inhalation of N-acetylcysteine<br>Group 1 will receive N-acetylcysteine inhalation every 12 hours from the beginning of invasive mechanical ventilation.
Active Comparator: Hypertonic saline<br>Inhalation of 5 ml-s of 5% sodium chloride every 12 hours.	Inhalation of 5% sodium chloride<br>Group 2 will receive inhalation with a 5% sodium chloride solution every 12 hours from the beginning of invasive mechanical ventilation.
Active Comparator: Bicarbonate<br>Inhalation of 5 ml-s 8.4% sodium bicarbonate every 12 hours.	Inhalation of 8,4% sodium bicarbonate<br>Group 3 will receive inhalation of 8.4% sodium bicarbonate every 12 hours from the beginning of invasive mechanical ventilation.
No Intervention: Control group<br>No preventive inhalation.	

Outcome Measures

Primary Outcome Measure:

1. Difference between 4 arms regarding ventilator-associated pneumonia rate
   Patients will be monitored for ventilator-associated pneumonia according to clinical criteria with a new or progressive pulmonary infiltrate on imaging plus supportive clinical findings of infection (eg, fever, secretions, leukocytosis). The diagnosis is confirmed when lower respiratory tract sampling identifies a pathogen.

   [Time Frame: Through study completion, an average of 6 months.]

Secondary Outcome Measure:

2. Number of ventilator-free days
   Number of days from day 1 after ICU admission and start of mechanical ventilation on which a patient breathes without assistance of the ventilator if the period of unassisted breathing lasted at least 24 consecutive hours.

   [Time Frame: Day 28 after ICU admission.]
3. Mortality
   Life status (alive or deceased) on day 28 after ICU admission.

   [Time Frame: Day 28 after ICU admission.]

Eligibility

Minimum Age:	18 Years
Maximum Age:	
Sex:	All
Gender Based:	No

Accepts Healthy Volunteers: No

Criteria: Inclusion Criteria:

- Patients over 18 years of age with coronavirus pneumonia who need invasive mechanical ventilation.

Exclusion Criteria:

- Patients with polytrauma,
- pregnant women,
- severe hemodynamic instability,
- patients with pulmonary edema,
- less than 3 days spent in the ICU,
- patients who have microbiologically proven bacterial infection on arrival.

Contacts/Locations

Central Contact Person: Nikola Delić, MD
Telephone: +385989000150
Email: nkldelic@gmail.com

Central Contact Backup:

Study Officials:

Locations: **Croatia**
Clinical Hospital Centre Split
Split, Croatia, 21000
Contact: Nikola Delić, MD +385989000150 nkldelic@gmail.com

IPDSharing

Plan to Share IPD: Undecided

References

Citations:

Links:

Available IPD/Information:
