# Supplementary material for: Effects of Different Inhalation Therapy on Ventilator-Associated Pneumonia in Ventilated COVID-19 Patients: A Randomized Controlled Trial
Source: Microorganisms. 2022 May 28;10(6):1118. doi: 10.3390/microorganisms10061118 (PMC9228146; doi:10.3390/microorganisms10061118)
Supplement: Supplementary file 1 [file microorganisms-10-01118-s001.zip › microorganisms-1662466-supplementary/Supplementary Material.pdf]

Supplementary Material

Supplementary Table S1. Comparison of laboratory parameters between the study groups.

Variables	Inhalation Type				*p*-Value *
	Control Group - No Inhalation (n=52; 29.7%)	N-Acetylcysteine Inhalation (n=39; 22.3%)	5% Saline Inhalation (n=42; 24.0%)	8.4% Sodium Bicarbonate Inhalation (n=42; 24.0%)	
**pH (initial)**	7.45 (7.44-7.50)	7.46 (7.42-7.52)	7.45 (7.43-7.49)	7.48 (7.45-7.50)	0.622
**pCO_2 (kPa; initial)**	4.5 (4.1-5.6)	4.6 (4.1-5.8)	4.7 (4.2-5.1)	4.4 (4.3-4.8)	0.344
**pO_2 (kPa; initial)**	5.9 (5.5-6.8)	6.7 (5.9-7.6)	6.4 (5.1-7.9)	6.5 (5.9-7.5)	0.486
**SpO_2 (%; initial)**	82.8 (78.2-88.7)	82.9 (79.9-89.4)	86.5 (75.7-89.5)	86.1 (81.5-90.4)	0.583
**HCO_3 (mmol/l; initial)**	25.3 (23.7-27.6)	24.6 (22.0-28.6)	24.5 (22.0-26.0)	25.8 (23.1-27.8)	0.316
**Base excess (initial)**	1.0 (-0.9-5.0)	1.3 (-1.7-5.9)	-0.5 (-2.2-3.8)	3.4 (-1.8-4.3)	0.546
**hsTnT (maximal)**	20.4 (14.5-39.4)	14.5 (9.8-66.1)	17.4 (11.8-25.6)	18.5 (10.8-38.6)	0.555
**NTproBNP (maximal)**	848.0 (399.0-2003.5)	1142.0 (353.0-1726.5)	567.0 (251.3-1254.8)	633.5 (281.0-4490.0)	0.406
**Red blood cells ($x10^{12}$)**	4.5 (4.2-4.8)	4.0 (3.8-4.5)	4.6 (4.3-4.9)	4.5 (4.1-5.3)	0.288
**Hemoglobin (g/l)**	136.0 (121.5-148.5)	116.5 (108.3-132.3)	131.0 (124.5-149.8)	130.5 (122.5-153.5)	0.099
**MCV (fl)**	90.3 (87.7-92.3)	89.5 (84.4-91.3)	87.1 (84.5-91.2)	90.6 (86.6-94.4)	0.652
**RDW**	13.9 (13.2-14.5)	13.7 (13.4-14.6)	13.5 (12.9-13.8)	13.7 (13.0-14.2)	0.537
**Platelets ($x10^9$)**	214.0 (165.5-286.0)	331.0 (143.3-456.3)	275.5 (202.5-339.3)	262.5 (222.3-323.3)	0.201

**WBC (x10^9)**	9.3 (7.1-13.0)	8.3 (5.3-13.9)	9.3 (6.9-11.8)	9.1 (7.3-14.2)	0.188
**Neutrophiles (x10^9)**	8.0 (5.9-11.4)	6.8 (4.5-12.8)	7.5 (5.3-10.5)	7.8 (5.1-16.0)	0.372
**Lymphocytes (x10^9)**	0.6 (0.3-0.8)	0.6 (0.4-0.9)	0.6 (0.4-1.1)	0.5 (0.4-0.9)	0.763
**CRP (mmol/l; initial)**	93.4 (47.6-116.0)	99.3 (53.3-156.3)	78.9 (60.4-127.0)	77.2 (50.2-135.51)	0.080
**CRP (mmol/l; maximal)**	222.5 (137.2-330.3)	228.0 (153.5-288.8)	160.9 (98.5-260.6)	183.1 (151.6-257.4)	0.666
**BUN (mmol/l; initial)**	9.6 (7.3-12.7)	9.3 (5.4-14.8)	8.4 (7.6-10.3)	9.1 (6.7-13.8)	0.768
**Creatinine (umol/l; initial)**	90.0 (76.5-112.0)	66.5 (46.8-107.0)	79.0 (71.0-91.8)	82.0 (62.3-151.3)	0.593
**eGFR (ml/min/1.73 m^2; initial)**	73.9 (52.1-87.0)	79.1 (58.2-101.4)	86.6 (70.2-91.6)	84.0 (41.7-98.1)	0.723
**AST (mmol/l; initial)**	53.0 (38.5-92.0)	43.0 (35.3-60.3)	40.0 (32.3-70.3)	56.5 (37.3-75.3)	0.318
**ALT (mmol/l; initial)**	52.0 (31.0-72.5)	42.5 (23.8-52.0)	37.0 (24.3-55.0)	43.5 (23.8-61.3)	0.318
**GGT (mmol/l; initial)**	64.0 (36.0-113.0)	55.0 (31.0-100.0)	66.5 (29.5-82.8)	41.0 (24.3-75.3)	0.190
**LDH (mmol/l; initial)**	548.0 (374.5-648.5)	528.0 (431.5-718.3)	486.0 (415.3-634.0)	589.0 (447.5-713.0)	0.580
**D-dimers (mmol/l; initial)**	1.4 (0.8-5.0)	1.6 (0.7-10.4)	1.3 (0.8-5.7)	7.7 (2.1-16.6)	0.223
**INR (units)**	1.0 (0.9-1.1)	1.0 (0.9-1.1)	1.0 (0.9-1.1)	1.0 (1.0-1.2)	0.390
**Sodium (mmol/l)**	138.0 (134.0-140.5)	139.5 (137.0-142.3)	137.5 (133.0-141.8)	139.0 (137.8-141.8)	0.773
**Chloride (mmol/l)**	100.0 (92.5-104.0)	104.5 (99.8-106.0)	100.5 (95.5-103.8)	102.0 (98.8-105.0)	0.728
**Potassium (mmol/l)**	4.3 (3.9-4.8)	4.1 (3.6-4.5)	4.4 (3.9-4.6)	4.1 (3.8-4.7)	0.852
**Calcium (mmol/l)**	2.0 (1.9-2.1)	2.0 (1.7-2.0)	1.9 (1.9-2.0)	1.9 (1.9-2.0)	0.797
**Phosphorus (mmol/l)**	1.0 (0.8-1.3)	1.1 (0.7-1.3)	1.0 (0.9-1.2)	1.3 (0.8-1.5)	0.712
**Protein (g/l)**	57.0 (53.0-63.0)	54.5 (51.5-58.3)	58.5 (54.0-62.3)	57.5 (53.8-63.0)	0.182
**Albumin (g/l)**	29.2 (28.1-32.0)	27.5 (25.1-32.1)	30.2 (29.9-32.9)	29.9 (27.1-32.4)	0.154

**Glucose (mmol/l)**	10.4 (7.2-13.3)	8.1 (6.4-12.2)	9.8 (7.2-13.9)	7.9 (7.5-10.9)	0.483

Data are expressed as number (percent) or median (interquartile range). * Kruskal Wallis test. **Abbreviations:** ALT – alanine aminotransferase; AST – aspartate aminotransferase; BUN – blood urea nitrogen; CRP – C-reactive protein; eGFR – estimated glomerular filtration rate; LDH – lactate dehydrogenase; GGT – gamma glutamyl transferase; HCO_3 – bicarbonate; INR – international normalized ratio; hsTnT – high-sensitivity troponin; MCV – mean corpuscular volume; pO_2 – oxygen partial pressure; pCO_2 – carbon dioxide partial pressure; SpO_2 – oxygen saturation; RDW – red cell distribution width; WBC – white blood cells.

Supplementary Table S2. Predictors of any fungal isolation.

Variables	Univariate Analysis		Multivariate Analysis	
	OR (95% CI)	*p*-Value	aOR (95% CI)	*p*-Value
**Age**	1.03 (0.99-1.06)	0.122	1.02 (0.97-1.07)	0.489
**Female sex**	1.95 (0.96-3.93)	0.064	2.29 (0.87-6.04)	0.093
**Duration of ventilator therapy (days)**	1.04 (1.01-1.07)	0.015	1.05 (1.01-1.10)	0.028
**Hospitalization duration (days)**	1.02 (1.00-1.04)	0.063	0.99 (0.97-1.02)	0.413
**Charlson Comorbidity Index**	1.11 (0.92-1.35)	0.290	0.92 (0.67-1.28)	0.628
**Prior smoking**	0.62 (0.26-1.47)	0.276	0.92 (0.33-2.57)	0.877
**Albumin (g/l)**	1.02 (0.93-1.12)	0.655	0.98 (0.88-1.10)	0.78
**Glucose (mmol/l)**	1.01 (0.95-1.08)	0.663	1.03 (0.94-1.12)	0.565

Abbreviations: OR – odds ratios; aOR – adjusted odds ratios.

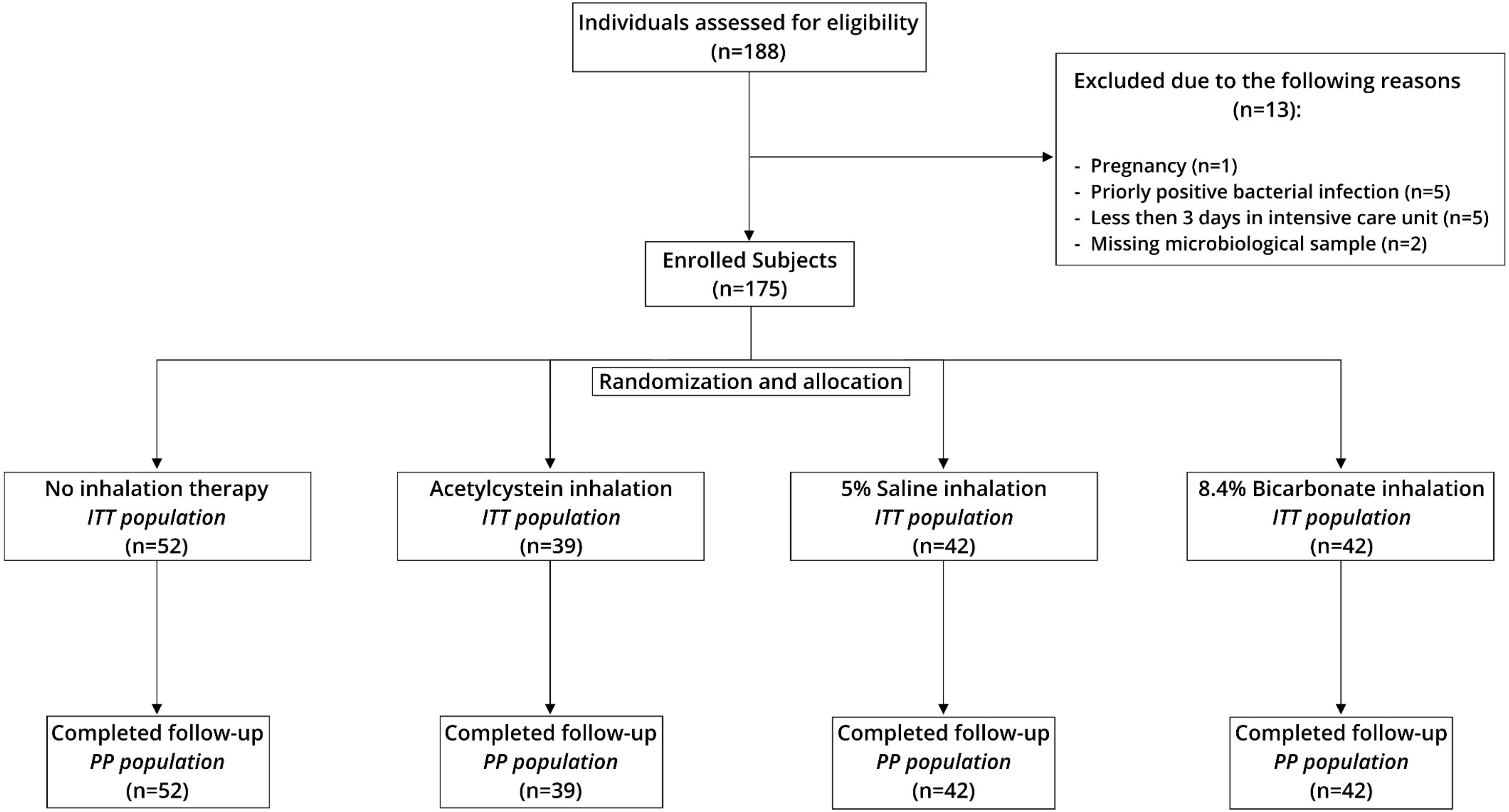

Supplementary Figure S1. Flow diagram. Abbreviations: none.
